# Maxillary incisors and molars with conventional and skeletal anchorage device: A randomized controlled trial

**DOI:** 10.6026/973206300210669

**Published:** 2025-04-30

**Authors:** Nirupama Rayast, Virendra Vadher, Shalabh Baxi, Shweta Singh, Barapatre Barapatre, Gangesh B. Singh

**Affiliations:** 1 Department of Orthodontics and Dentofacial Orthopedics, Government Dental College, Raipur, Chhattisgarh, India

**Keywords:** Orthodontic anchorage procedures, dental space closure, malocclusion, cephalometry, mini-implant

## Abstract

Effect of traditional anchorage techniques and new mini-implant methods on the positioning of maxillary incisors and molars in terms of anteroposterior, vertical and angular changes is of interest. Hence, eighteen patients, with either Angle Class II Division 1 malocclusion or Class I malocclusion with a bimaxillary protrusion, were split into two groups: Group 1, which used mini-implants and Group 2, which relied on conventional anchorage. Space was closed within 12-18 months and the lateral cephalograms and orthopantomograms (OPGs) were taken at the start (T1) and after space closure (T2). In Group 1, significant movement of the maxillary first molars in both a distal and intruding direction into the bone was observed. Meanwhile, the molars in Group 2 were shifted mesially and extruded. Molar tipping was more stable in Group 1. Mini-implant anchorage proved more efficient in controlling molar movement, retraction and anterior teeth tipping.

## Background:

A crucial concern in orthodontic therapy is the regulation of anchorage, which mitigates adverse effects while facilitating desirable enamel movements, particularly during space closure [1, 2]. Establishing effective anchorage is challenging due to weakened periodontal tissues in individuals with adult periodontitis [3]. Managing this malocclusion often necessitates the extraction of maxillary (or bimaxillary) first or second premolars while optimising anchorage [4]. Various auxiliaries can be employed to enhance anchorage, such as a Nance holding arch, helmet, trans-palatal arch and others [5]. Nonetheless, all these methods present intrinsic drawbacks, including complex designs, the need for exceptional patient compliance and precise wire manipulation [6]. Consequently, some loss of anchorage or mesial displacement of the maxillary molars is commonly observed. Despite the widespread use of traditional anchorage methods, including intraoral appliances and extraoral devices, they frequently encounter limitations, such as the risk of anchorage loss and reduced patient compliance [7, 8]. Temporary skeletal anchorage devices facilitate orthodontic movements that were previously considered challenging, if not unfeasible [9]. The introduction of bone anchorage devices, particularly mini-implants, has enabled alternative strategies for improved stability and management. In recent years, mini-implants have gained considerable traction within the orthodontic community [10]. The effectiveness of mini-implants in the assessment of standard anchorage strategies has been examined in current studies [11, 12-13]. Mini-implants are very powerful at improving intraoral anchorage during the retraction and small intrusion of the upper front enamel, consistent with clinical cephalometric research by Upadhyay *et al.* [14] Anchorage is maintained in both the horizontal and vertical instructions. Al-Sibaie *et al.* [15] additionally found that using mini-implant anchorage for en-masse retraction caused a more balanced movement and a mild incursion of the upper front teeth of their randomised controlled have a look at. Traditional techniques, then again, ended in a few managed enamel tilting. These findings mean that we may additionally have extra management over tooth motion at some point of space closure using mini-implants. Despite these developments, greater comparison research is nevertheless required to identify an appropriate positional shift of maxillary incisors and molars, while the use of mini-implants is antagonistic to traditional anchorage gadgets [16]. Knowing these differences is essential for optimising treatment planning and consequences in orthodontic exercise. Therefore, it is of interest to evaluate the changes within the function of maxillary incisors and molars during space closure with the use of traditional anchorage and skeletal anchorage gadgets.

## Methods and Materials:

The study was set up as a Randomized Controlled Trial (RCT) in the Department of Orthodontics and Dentofacial Orthopedics at Government Dental College in Raipur. Before beginning, we were given ethical approval with IEC Proposal No. 4736/GDC/ETHICS COMMITTEE/2022 and all contributors provided knowledgeable written consent. Those who met the inclusion standards were randomly divided into groups: Group 1, wherein mini-implants (1.5x8 mm) were used for skeletal anchorage throughout en-masse retraction and Group 2, where conventional anchorage with an elastomeric E-chain for remaining spaces was used. Patients requiring orthodontic treatment that involves the extraction of maxillary first premolars and who maintain suitable oral hygiene as part of their commitment to the treatment plan were included. Patients with systemic conditions affecting bone metabolism, a record of preceding orthodontic treatment, or craniofacial anomalies were excluded. A standardised protocol for both clinical and radiographic tests was followed and digital lateral cephalograms and orthopantomograms using the Planmeca Proline XC Dimax 3 Ceph were used to evaluate changes before and after treatment. The tracing was done on acetate paper with a 0.3 mm HB lead pencil using an illuminator, focusing on specific anatomical landmarks like Sella (S), Nasion (N) and Anterior Nasal Spine (ANS) to assess the movements of the maxillary incisors and molars in different directions. In Group 1, the mini-implant anchorage group, we placed titanium mini-screws (1.5 x 8 mm) between the maxillary second premolar and first molar on both sides, all under local anaesthesia. A crimpable hook was positioned behind the lateral incisors and we did the retraction using a closed nickel-titanium coil spring that applied 150 grams of force on each side. We loaded the mini-implants one week after placement to ensure they were stable. For Group 2, the conventional anchorage group, we achieved retraction with an elastomeric E-chain attached to the molar tube hooks. Both groups used a 0.022 MBT slot appliance, starting with a levelling and alignment phase using a 17x25 stainless steel archwire before closing the spaces. All instruments were thoroughly sterilised using an autoclave and patients received a preventive antibiotic about an hour before the procedure. They were asked to rinse their mouths with a 0.2% chlorhexidine mouthwash and we disinfected their upper and lower lips with Betadine solution before placing the mini-implants. We systematically collected data and analysed cephalometric tracings to observe any changes in the positions of molars and incisors before and after treatment, using various reference points and planes.

## Statistical analysis:

SPSS software was used to evaluate the results, looking for significance with a p-value set at less than 0.05. We compared the treatment outcomes between the two groups using appropriate statistical tests to assess how effective skeletal anchorage was compared to conventional anchorage methods for controlling space closure movements.

## Results:

In our results, we looked at how the positions of maxillary incisors and molars changed during space closure with two different anchorage techniques: conventional anchorage (G2) and skeletal anchorage with mini-implants (G1). We conducted cephalometric analyses at two key points-before treatment (T1) and after treatment (T2)-to evaluate horizontal, vertical and angular changes. For maxillary molars, looking at the horizontal movement, G1 showed a slight backward shift of -0.67 ± 0.53 mm at the mesiobuccal cusp tip and -0.87 ± 0.62 mm at the distobuccal cusp tip. On the other hand, G2 had a forward movement of 2.3 ± 1.3 mm and 1.83 ± 1.26 mm at the same points. These differences were quite significant. Vertically, G1 demonstrated a downward movement of -0.65 ± 0.6 mm and -0.6 ± 0.41 mm, while G2 showed an upward movement of 1.04 ± 1.68 mm and 1.033 ± 0.87 mm, with significant differences noted here as well. In terms of angular movements, G1 had a distal crown tip of -1.2° ± 1.13°, while G2 had a mesial crown tip of 2.5° ± 3.9°, indicating a notable difference in how the molars tipped between the two groups. For maxillary incisors, in the horizontal plane, the G1 group (using mini-implants) showed a root movement of -1.15 ± 1.69 mm and a crown movement of -5.2 ± 2.7 mm. Meanwhile, the G2 group (using conventional anchorage) had a root movement of 0.43 ± 3.36 mm and a crown movement of -2.35 ± 3.7 mm. However, these differences weren't statistically significant, suggesting that both anchorage methods had similar effects on incisor retraction. Vertically, G1 had a greater incisor root intrusion at -1.733 ± 2.23 mm and crown extrusion at 2.20 ± 1.3 mm compared to G2, which showed minimal changes. Still, these differences weren't significant, indicating that both groups kept a similar vertical control. When looking at angular changes, G1 experienced more incisors tipping at -11.1° ± 6.50°, while G2 showed a tipping of -3.44° ± 6.04°. This difference was significant, suggesting that mini-implants offered better control and effectiveness in preventing excessive tipping during retraction. You can find the detailed cephalometric changes in maxillary incisors and molars in Table 1. Cephalometric analyses were performed at two time points-T1 (pre-treatment) and T2 (post-treatment)-to evaluate horizontal, vertical and angular changes. A comparison of pre- and post-treatment mean values for conventional anchorage (G2) and skeletal anchorage with mini-implants (G1) is shown in Figure 1 (see PDF).

## Maxillary first molar measurements:

[1] U6M-SV- distance from the greatest mesial convexity on the first molar to the SV line (Perpendicular to SN plane through point S on the skull).

[2] U6D-SV- distance from the greatest distal convexity on the first molar to SV line (Perpendicular to SN plane through point S on the skull). 

[3] 6M-PP- distance from the greatest mesial convexity on the first molar to the Palatal Plane.

[4] U6D-PP- distance from the greatest distal convexity on the first molar to the Palatal Plane. 

[5] U6 angle-angle formed by the longitudinal axis of the molar and the SV line (Perpendicular to SN plane through point S on the skull).

## Maxillary central incisor measurements:

[1] Io-SV- distance between the incisal edge of the maxillary central incisors and the SV line (Perpendicular to SN plane through point S on the skull).

[2] Ia-SV- distance between the apical tip of the root of the maxillary central incisors and the SV line (Perpendicular to SN plane through point S on the skull).

[3] Io-PP- distance between the incisal edge of the maxillary central incisors and the Palatal Plane.

[4] Ia-PP- distance between the apical tip of the root of the maxillary central incisors and the Palatal Plane.

[5] I angle-angle between the longitudinal axis of the maxillary central incisor and the SN plane.

## Discussion:

The present study evaluated the positional modifications of maxillary incisors and molars through area closure using two special anchorage strategies: skeletal anchorage with mini-implants (G1) and conventional anchorage (G2). The results demonstrated that mini-implants provided advanced anchorage management, minimizing molar mesial movement and maintaining higher vertical and angular control in comparison to traditional anchorage.

## Maxillary molar movements:

The examination showed a few outstanding differences in how the maxillary molars moved between the two groups. In the horizontal direction, the group using mini-implants (G1) had a controlled motion of the molars, with shifts of approximately -0.67 mm at the mesiobuccal cusp tip and -0.87 mm at the distobuccal cusp tip. In contrast, the conventional anchorage organisation (G2) showed a significant movement in the direction of the front, measuring 2.3 mm and 1.83 mm at the identical factors. These results were very large (P < 0.001), highlighting how effective mini-implants are in maintaining the molars in the region. Similar results were stated in Thiruvenkatachari *et al.* [16] and Deguchi *et al.* [17], highlighting the function of skeletal anchorage in minimising anchorage loss. Vertically, G1 exhibited molar intrusion (-0.65 ± 0.6 mm and -0.6 ± 0.41 mm), whereas G2 showed molar extrusion (1.04 ± 1.68 mm and 1.033 ± 0.87 mm), with statistically significant differences (P < 0.05). This finding helps the usage of mini-implants in preventing undesired vertical molar motion, which may be important in instances requiring absolute anchorage, which is analogous in Tseng *et al.* [18]. Angular changes further highlighted the benefits of mini-implants. G1 demonstrated distal molar tipping (-1.2° ± 1.13°), whereas G2 experienced mesial tipping (2.5° ± 3.9°) (P < 0.01). These findings align with Papadopoulos *et al.* [19] and Park *et al.* [20] studies indicating that mini-implants effectively control unwanted molar tipping and provide better force distribution during space closure.

## Maxillary incisor movements:

Regarding incisor retraction, both groups exhibited significant crown movement; however, differences between them were not statistically significant (P > 0.05). The root retraction in G1 (-1.15 ± 1.69 mm) and G2 (0.43 ± 3.36 mm) suggested that mini-implants might offer better control, though the variation within groups limited statistical significance. Similar trends have been noted in the study by Kuroda *et al.* [21], where mini-implants provide enhanced control over anterior retraction but may still allow some variability in movement. Vertically, G1 exhibited greater root intrusion (-1.73 ± 2.23 mm) and crown extrusion (2.20 ± 1.31 mm) compared to G2 (-0.14 ± 1.71 mm and 0.10 ± 1.87 mm, respectively), but these differences were not statistically significant (P > 0.001). This suggests that while mini-implants may provide better incisor vertical control, variations in patient response and biomechanics can influence outcomes, a similar finding with Upadhyay *et al.* [14]. In another study by Jayaratne *et al.* it was noted that incisor retraction was more pronounced with buccally positioned mini-implants compared to traditional anchorage methods. Nonetheless, the retraction of incisors was diminished with indirect anchorage from palatal mini-implants in comparison to buccally positioned mini-implants [22]. Angular changes in incisor inclination revealed a significantly greater degree of incisor tipping in G1 (-11.1° ± 6.50°) compared to G2 (-3.44° ± 6.04°) (P < 0.05). This finding aligns with the results of the study by Lin *et al.* [23], which demonstrated that mini-implants facilitate bodily incisor retraction while reducing uncontrolled tipping associated with conventional anchorage. The reduced tipping observed in the mini-implant group may contribute to improved esthetic and functional outcomes in orthodontic space closure procedures.

## Clinical implications:

The findings of this study reinforce the advantages of skeletal anchorage in orthodontic space closure. Mini-implants provide superior control of molar anchorage, limiting mesial movement, preventing vertical extrusion and minimising undesired tipping. Although incisor retraction was comparable between groups, mini-implants demonstrated superior control over incisor inclination, thereby optimising biomechanics during treatment.

## Limitations and future directions:

While this study has its strengths, there are a few limitations worth mentioning. The sample size is decent; however, it could be larger in future studies to make the findings more applicable to a wider target audience. Additionally, variations in affected person reaction to remedy mechanics and man or woman anatomical differences may impact results. Future research should explore the long-term balance of area closure carried out with mini-implants and assess patient-stated effects to recognise their scientific impact better.

## Conclusion:

Data shows that mini-implants provide improved management over anchorage and tooth motion, ensuring predictable and green treatment outcomes. Mini-implants can minimise unwanted facet consequences, such as anchor loss and deep bite formation and lower usual remedy times and they provide tremendous benefits for both patients and orthodontists.

## Declarations:

## Author contribution:

All authors contributed extensively to the study. Nirupama Rayast conceptualised the research, designed the technique and led data collection. Shalabh Baxi supervised the examination, tested the method and executed statistical analysis. Shweta Singh performed the literature assessment, interpreted records and contributed to manuscript writing. Nirupama Rayast and Chhaya were liable for clinical data collection, affected person management and surgical approaches. Gangesh B. Singh handled record analysis, organized figures and tables and ensured adherence to ethical recommendations. Virendra Vadher conducted the final assessment, edited the manuscript and facilitated investment acquisition. All authors reviewed and accepted the very last version of the manuscript.

## Funding:

None

## Institutional review board statement:

Ethical approval was acquired before initiating the study with IEC Proposal No. 4736/GDC/ETHICS COMMITTEE/2022.

## Informed consent statement:

Informed written consent was acquired from all participants.

## Figures and Tables

**Table 1:** Cephalometric changes in maxillary incisors and molars

**Measurement**	**G1 (Mini-Implant Group)**	**G2(Conventional Anchorage)**	**P-value**
Molar Distal Movement (mm)	-0.67 ± 0.53	+2.3 ± 1.3	<0.001**
Molar Vertical Movement (mm)	-0.65 ± 0.6	+1.04 ± 1.68	<0.05*
Molar Angular Change (°)	-1.2 ± 1.13	+2.5 ± 3.9	<0.01**
Incisor Root Retraction (mm)	-1.15 ± 1.69	+0.43 ± 3.36	>0.05
Incisor Crown Retraction (mm)	-5.2 ± 2.7	-2.35 ± 3.7	>0.05
Incisor Intrusion (mm)	-1.73 ± 2.23	-0.14 ± 1.71	>0.001
Incisor Tipping (°)	-11.1 ± 6.50	-3.44 ± 6.04	<0.05*
Statistical Significance: *P <
0.05- significant; **P <
0.01- highly significant;
***P <
0.001- very highly significant.
